# Supplementary material for: Exhausted-like effector CD8 T cells mediate immune-stromal interactions at mucosal Chronic Graft-versus-Host Disease onset
Source: bioRxiv. 2025 Sep 16:2025.09.11.675642. Preprint. [Version 1] doi: 10.1101/2025.09.11.675642 (PMC12458946; doi:10.1101/2025.09.11.675642)
Supplement: Supplement 1 [file media-1.pdf]

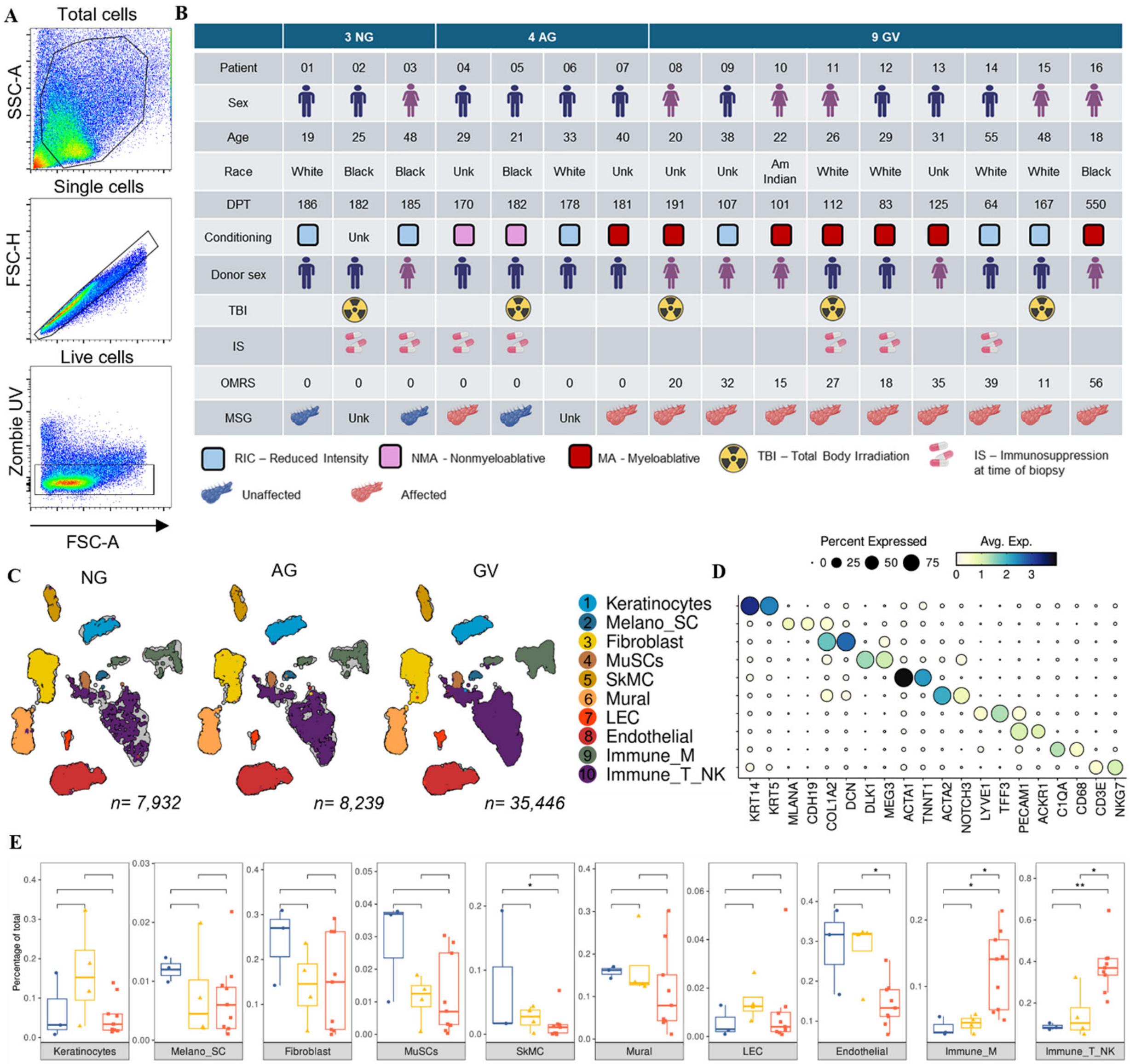

Supplemental Figure 1: Clinical metadata and cellular composition of oral mucosa in NG, AG, and GV groups. (A) Flow cytometry gating strategy for isolating viable (Zombie^{-}) CD45^{+} immune cells from oral mucosa (OM) biopsies. **(B)** Clinical and demographic characteristics of patients included in the single-cell RNA sequencing analysis. Columns indicate individual patient metadata including gender, age, race, transplant conditioning regimen (RIC: blue, NMA: pink, MA: red), total body irradiation (TBI), donor gender, immunosuppressive treatment (IS), oral cGVHD clinical score (OMRS), and minor salivary gland involvement (MSG). OMRS > 0 indicates clinical oral cGVHD (GV), while AG patients have histological evidence without clinical symptoms. DPT: Days post-transplant. **(C)** UMAP projections of integrated OM single-cell data from NG (n = 7,932 cells), AG (n = 8,239), and GV (n = 35,446) patients, colored by cell type identity (as defined in Fig. 1F). **(D)** Dot plot showing key marker genes used to annotate the 10 identified OM cell types. Dot size indicates the percentage of cells expressing the gene within each

cluster; color intensity reflects average expression level. **(E)** Boxplots showing relative frequencies of each cell type across NG (blue), AG (yellow), and GV (red) samples. Significant increases in myeloid and lymphoid populations are observed in GV patients. Each dot represents one patient sample; data shown as mean ± SEM. Statistical comparisons by two-sided Wilcoxon test; $^*p \leq 0.05$ considered significant.

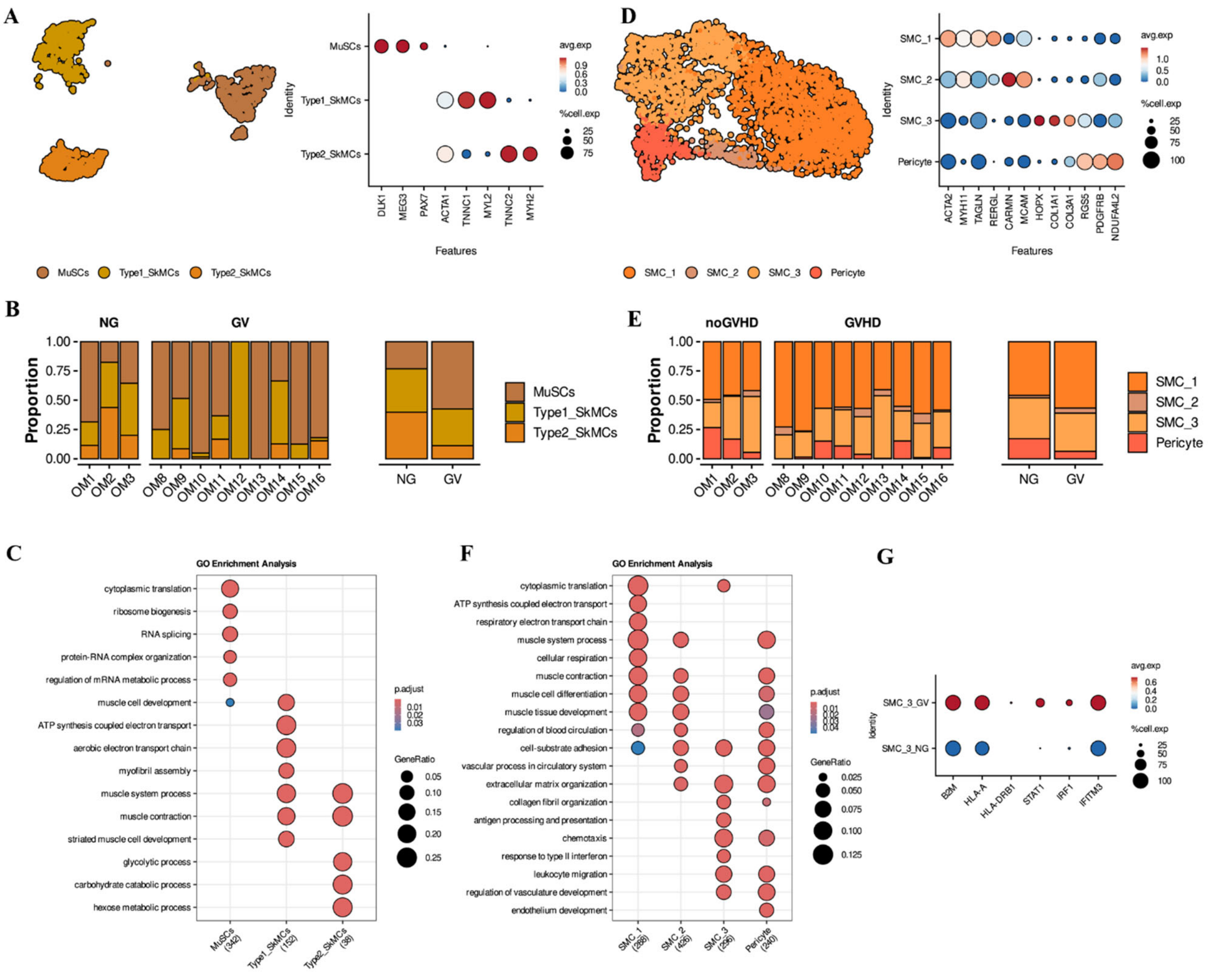

Supplemental Figure 2. Expanded analysis of non-immune cell subsets: Muscle and Smooth Muscle Cell Alterations in Oral cGVHD. (A) UMAP (left) of integrated muscle cell populations and dot plot (right) showing expression of representative marker genes for each subcluster **(B)** Bar plots showing the proportion of muscle cell subclusters per individual patient (left) and by group (NG vs. GV, right). Significant differences assessed by two-sided paired Wilcoxon test; $p < 0.05$. Data was assessed by two-sided paired Wilcox test, and p-values < 0.05 was considered statistically significant. **(C)** GO Biological Process (GO BP) pathway enrichment analysis of genes upregulated in each muscle subcluster. Circle size indicates gene ratio; color scale reflects adjusted p-value (Benjamini–Hochberg). **(D)** UMAP (left) and corresponding dot plot (right) of smooth

muscle cell (SMC) clusters showing signature gene expression across subpopulations. **(E)** Cell proportion bar plots of SMC subclusters per patient (left) and grouped by NG vs. GV (right). Differences notated as in panel (B). **(F)** GO BP enrichment for genes upregulated in smooth muscle cell subclusters. Significance assessed using one-sided Fisher's exact test with BH correction. **(G)** Dot plot of differentially expressed genes in SMC_3 between NG and GV patients. Dot size reflects percentage of expressing cells; color denotes scaled average expression.

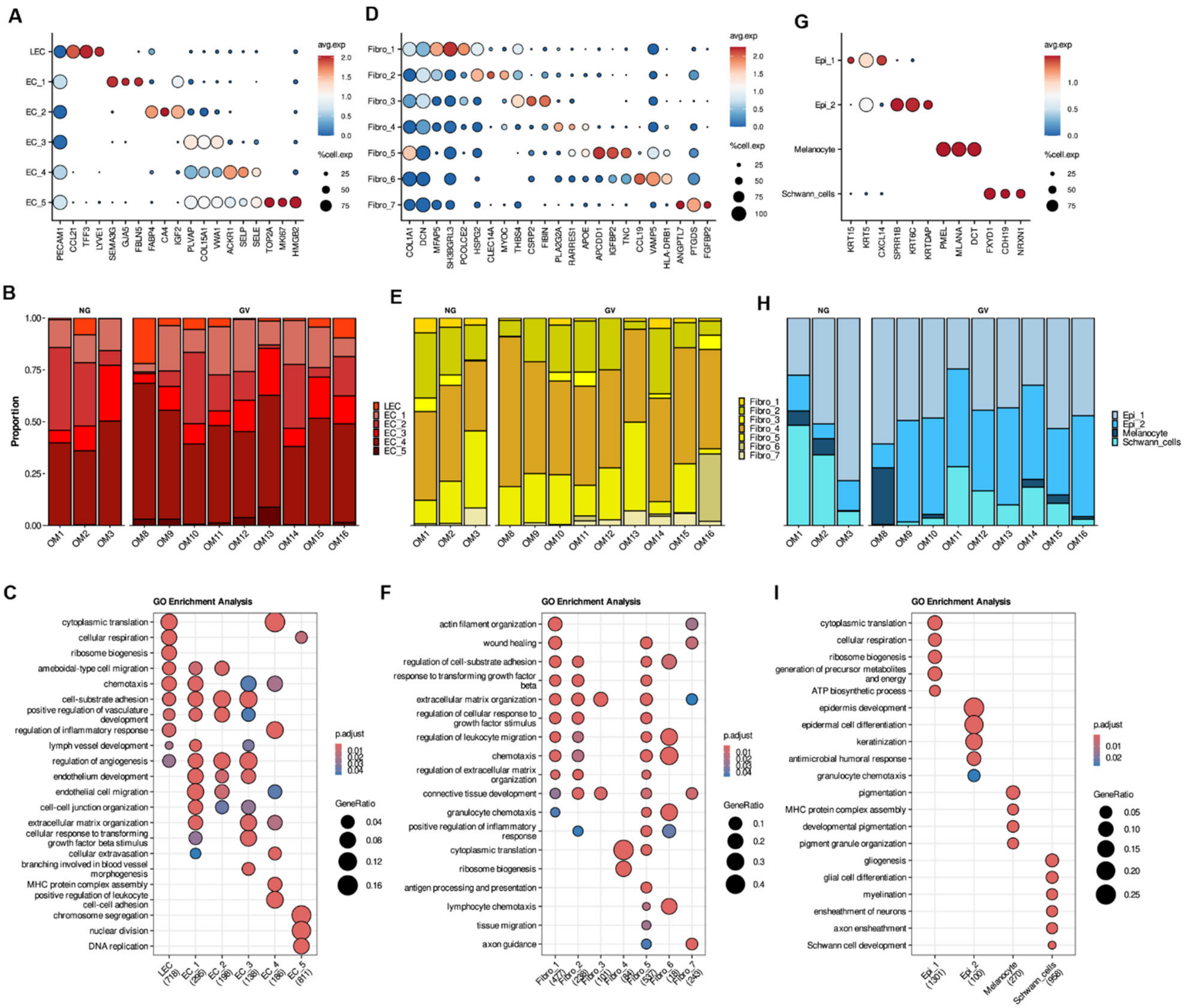

Supplemental Figure 3. Expanded analysis of non-immune cells: Stromal and epithelial reprogramming in oral cGVHD. (A) Dot plot of signature genes across endothelial subclusters, illustrating functional and phenotypic heterogeneity. **(B)** Proportional distribution of endothelial subclusters per individual patient. **(C)** GO Biological Process (GO BP) pathway enrichment analysis of pathways upregulated in endothelial subpopulations. Circle size indicates gene ratio; color scale reflects adjusted *p*-value (Benjamini–Hochberg). **(D)** Dot plot showing marker gene expression across fibroblast subclusters. **(E)** Cell proportions of fibroblast clusters per patient. **(F)** GO BP enrichment analysis for upregulated genes in fibroblast subpopulations. **(G)** Dot plot showing the expression of signature genes across epithelial clusters. **(H)** Bar plot of epithelial subcluster proportions per patient. **(I)** GO BP enrichment for epithelial subclusters. Size of each

circle represents gene ratio, and color indicates adjusted *p*-value (one-sided Fisher's exact test with Benjamini–Hochberg correction).

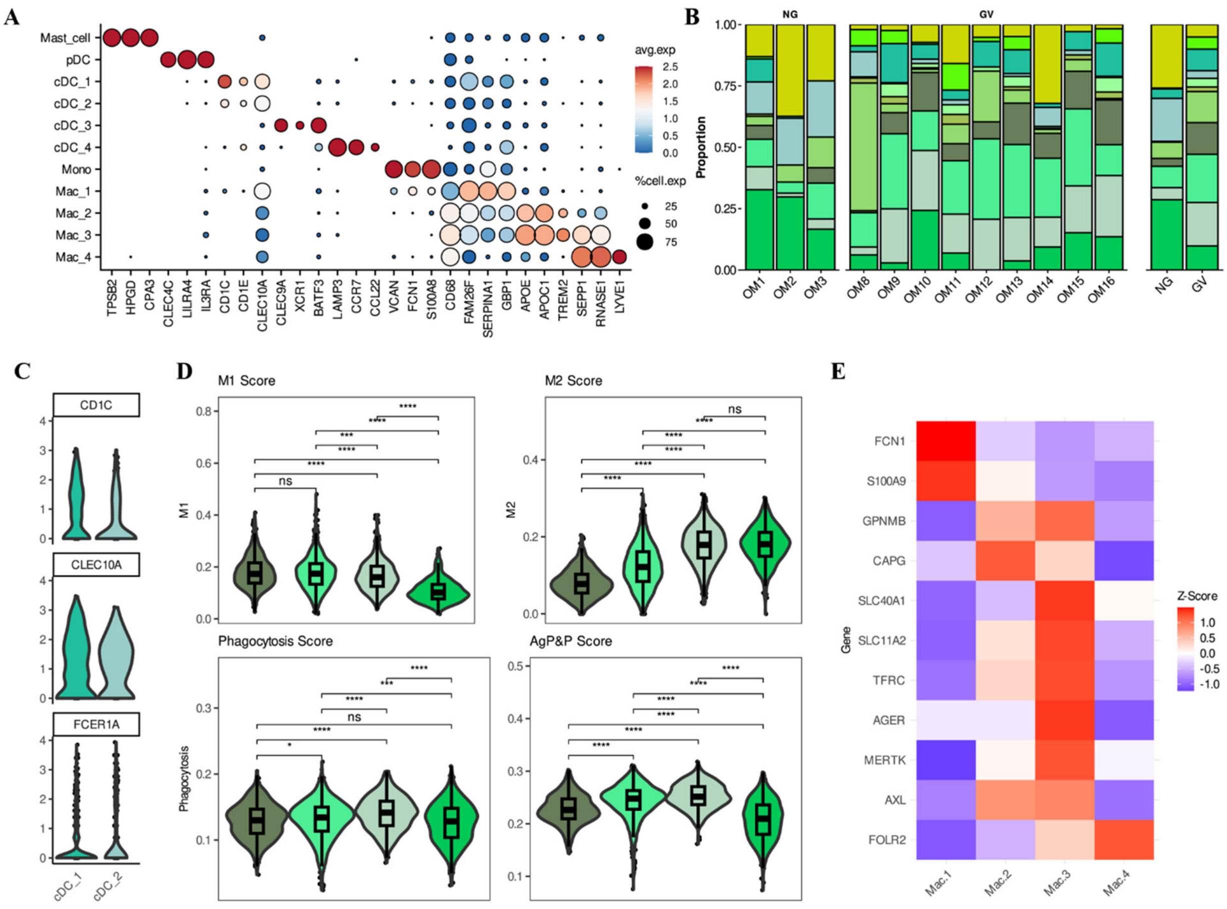

Supplemental Figure 4. Extended characterization of myeloid subsets in oral cGVHD. (A) Dot plot showing the expression of canonical marker genes used to annotate individual myeloid cell subsets, including dendritic cells, mast cells, monocytes, and macrophages. Dot size reflects the proportion of expressing cells; color intensity indicates average scaled expression. **(B)** Bar plots showing the proportion of each myeloid cluster across individual patients (left) and grouped by disease status (NG vs. GV, right). **(C)** Violin plots illustrating the expression of representative genes distinguishing cDC_1 and cDC_2 subsets. **(D)** Violin plots of M1, M2, antigen processing and presentation (AgP&P), and phagocytosis gene signature AUC scores across macrophage subsets. Statistical comparisons were performed using the Wilcoxon rank-sum test. **(E)** Heatmap showing expression patterns of selected marker genes across macrophage clusters, highlighting phenotypic heterogeneity.

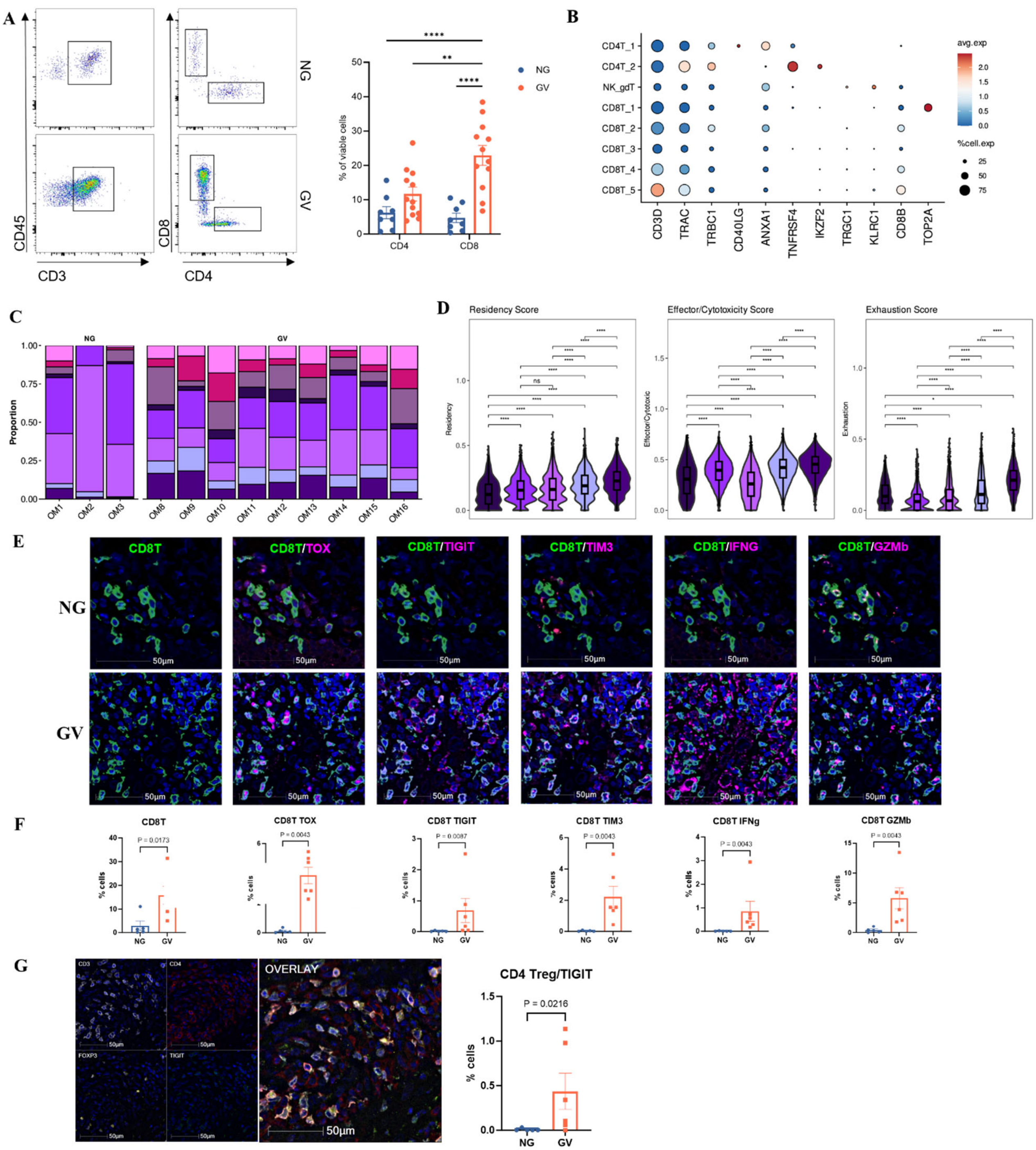

Supplemental Figure 5. Additional characterization of exhausted-like CD8$^+$ T cells and Tregs in oral cGVHD. (A) Representative flow cytometry gating strategy for CD4$^+$ and CD8$^+$ T cell analysis from oral mucosa biopsies (left). Quantification of CD4$^+$ and CD8$^+$ T cell frequencies in NG (n = 8) and GV (n = 12) patients. Each dot represents an individual sample; bars indicate mean ± SEM. Statistical analysis performed using Two-way ANOVA, Sidak's post-test. **(B)** Dot plot showing expression of key signature genes across annotated lymphoid cell clusters. Dot size

reflects the percentage of expressing cells; color intensity indicates scaled average expression. **(C)** Bar plots showing the relative proportions of lymphoid clusters across individual patients from NG and GV groups. **(D)** Violin plots display AUC scores for residency, cytotoxic/effector function, and exhaustion signatures across individual $CD8^+$ T cell clusters. Statistical significance assessed via Wilcoxon rank-sum test. **(E)** Representative IF images of OM tissue stained for CD8 (green), and exhaustion/cytotoxicity markers (TOX, TIGIT, TIM-3, IFNG, GZMB; magenta). DAPI (blue) marks nucleated cells. Scale bars = 50 µm. **(F)** Quantification of $CD8^+$ T cells and their co-expression with exhaustion/cytotoxicity markers in NG (n = 5) and GV (n = 6) tissues. Statistical significance determined by Wilcoxon rank-sum test. **(G)** Multiplex IF staining (left) of CD3 (white), CD4 (red), FOXP3 (yellow), and TIGIT (green) in oral mucosa tissue. DAPI (blue) indicates nuclei. Images acquired at 20× magnification; scale bars = 50 µm. Quantification (right) of $TIGIT^+$ $CD4^+FOXP3^+$ regulatory T cells (Tregs) in NG (n = 5) and GV (n = 6) samples. $p < 0.05$ considered statistically significant (Wilcoxon rank-sum test).

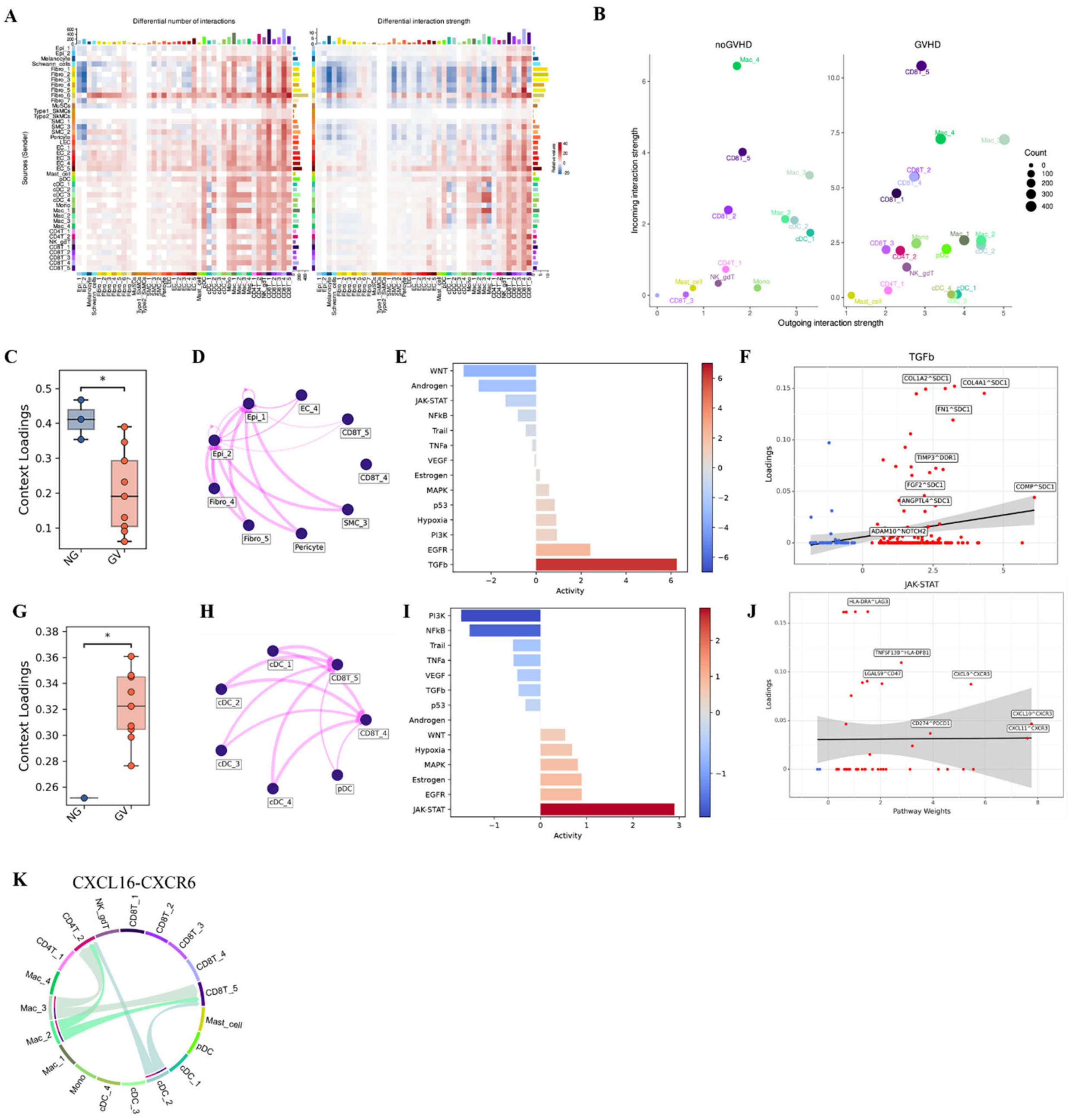

Supplemental figure 6: Additional insights into stromal and immune cell signaling in the GVHD-affected oral mucosa microenvironment. (A) Heatmaps showing the differential number (left) and strength (right) of predicted cell–cell interactions across cell types between NG and GV oral mucosa. **(B)** Scatter plots of outgoing versus incoming interaction strength per cell type, showing altered network topology in GV compared to NG patients. **(C)** Box plots compare downregulated context loadings between NG and GV patients related to stromal/epithelial-CD8$^+$

T cells interactions identified through Tensor-cell2cell analysis. Pairwise t-tests were performed, and p-values were adjusted using the Benjamini–Hochberg method. Statistical significance is indicated by asterisks ($p < 0.05$). **(D)** Network diagrams illustrate interactions between $CD8^{+}$ T cells and stromal/epithelial cells corresponding to (C), with connecting lines representing interaction strength. **(E)** Pathway enrichment analysis of ligand–receptor loadings identified using PROGENy. Color intensity reflects activity magnitude and reveals elevated TGF-β signaling activity in NG patients. **(F)** Scatter plots showing ligand–receptor pairs contributing to the TGF-β pathway enrichment shown in (E), with regression lines and confidence intervals (shaded areas) indicating trends and variability.

Table S1: Patient Demographics

Demographics	scRNA seq		
	NG	AG	GV
Number of cases	3	4	9
Age at sample, yr, median (range)	25 (19-48)	31 (21-40)	29 (18-55)
Sex (F/M)	1/2	0/4	5/4
Race (%)			
White	1 (33.3)	1 (25)	4 (44.5)
African	2 (66.7)	1 (25)	1 (11.1)
Asian			1 (11.1)
Hispanic			
Unknown		2 (50)	3 (33.3)
Time post-HSCT, days, median (range)	185 (182-186)	179.5 (170-182)	112 (64-550)
**Underlying Disease, n (%)**			
Lymphoma (HD/NHL)	1 (33.3)	2 (50)	1 (11.1)
Acute leukemia (AML/ALL)		1 (25)	4 (44.5)
Chronic leukemia (CLL/CML)			1 (11.1)
MDS/myeloproliferative disorder (MDS, myelofibrosis, PV)			2
Sickle cell disease	2 (66.7)	1 (25)	
Other			1 (11.1)
**Donor type, n (%)**			
Related	2 (66.7)	1 (25)	8 (88.9)
Unrelated	1 (33.3)	3 (75)	1 (11.1)
**HLA match, n (%)**			
Matched		1 (25)	3 (33.3)
Mismatched	1 (33.3)	1 (25)	
Haploidentical	2 (66.7)	2 (50)	6 (66.7)
**Sex match (donor -> recipient) , n (%)**			
F ® F	1 (33.3)		3 (33.3)
F ® M			2 (22.2)
M ® M	2 (66.7)	4 (100)	2 (22.2)
M ® F			2 (22.2)
Not Available			
**Conditioning regimen , n (%)**			
Myleoablative	1 (33.3)	1 (25)	4 (44.4)
Nonmyeloablative	2 (66.7)	3 (75)	5 (55.6)
History of TBI, n(%)	1 (33.3)	1 (25)	3 (33.3)
**Stem cell source , n (%)**			
PBSC	2 (66.7)	3 (75)	3 (33.3)
Bone Marrow	1 (33.3)	1 (25)	6 (66.7)
History of aGVHD, yes, n (%)	1 (33.3)	2 (50)	5 (55.6)
**Systemic immunosupressive treatment at time of saliva sample n (%)**			
None		2 (50)	6 (66.7)
Steroid			2 (22.2)
Calcineurin/mTOR inhibitor	3 (100)	1 (25)	1 (11.1)
Calcineurin/mTOR inhibitor + Steroid		1 (25)	
Other			

Table S2: 10X Chromium sequencing details

S.No.	Disease group	Estimated No. of cells	Fraction Reads in cells	Mean Reads/cell	Median genes/cell	Total Genes detected	Median UMI/cell
1	NG	2,782	62.50%	84,354	1,862	22,633	5,326
2	NG	5,177	71.40%	34,944	1,399	23,345	3,377
3	NG	1,955	76.70%	51,601	1,944	22,011	4,449
1	AG	5,158	76.50%	17,465	901	22,232	2,253
2	AG	4,096	56.70%	55,262	1,217	23,127	2,930
3	AG	3,212	76%	97,976	1,726	23,889	4,474
4	AG	992	60.30%	143,743	955	20,466	4,153
1	GV	8,135	78.50%	18,454	924	23,946	2,455
2	GV	2,266	54.60%	54,649	1,184	22,427	2,982
3	GV	2,833	83.80%	40,479	1,970	23,278	5,298
4	GV	2,499	76.20%	60,154	1,876	22,852	5,138
5	GV	1,059	75.30%	86,103	1,290	20,084	3,078
6	GV	10,379	80.90%	25,996	1,299	24,597	2,817
7	GV	3,058	59.40%	97,408	1,505	22,951	3,659
8	GV	2,269	59.70%	71,716	1,447	21,541	3,649
9	GV	10,995	75.60%	12,736	924	23,855	1,993

Table S3: Genes used to define cell state scores – non-immune cells (related to Figures 2D-F)

Antigen Presentation Score	Chemotaxis score	Regulation of Inflammatory Response
GOBP_ANTIGEN_PROCESSING_AND_PRESENTATION (GO:0019882)	GOBP_LEUKOCYTE_CHEMOTAXIS (GO:0030595)	GOBP_REGULATION_OF_INFLAMMATORY_RESPONSE (GO:0050727)

Table S4: Genes used to define cell state scores – myeloid cells (related to Figures 3H, I)

M1	M2	Phagocytosis	AgP&P
IL1B	CCL17	GOBP_PHAGOCYTOSIS (GO:0050766)	GOBP_ANTIGEN_PROCESSING_AND_PRESENTATION (GO:0019882)
IFNG	CCL22		
CD40	CCL24		
CD80	IL10		
CD86	TGFB1		
IL1R1	CCL13		
HLA-DRA	VEGFA		
TLR2	PDGFA		
TLR4	PDGFB		
IFNAR2	MMP9		
IFNAR1	VEGFB		
FCGR1A	TGFB2		
CCL3	TGFB3		
CCL4	MMP14		
CCL5	MMP19		
CXCL8	SEPP1		
CXCL9	IDO2		
CXCL10	CD163		
CXCL11	MSR1		
TNF	LYVE1		
IL6	STAB1		
IL1A	MARCO		
KYNU	CD36		
IL12B	FCGR2A		
SLC2A1	IL1R2		
TFEB	IL4R		
STAT1	CD274		
NFKB1	PDCD1LG2		
STAT3	PDCD1		
IFITM3	SIRPA		
IRF3	SIGLEC10		
IRF5	LILRB1		
IRF7	LILRB2		
SOCS3	TEK		
JUN	TREM1		
IDO1	TREM2		
IRF1	IL1RN		
	STAT6		
	MAFB		
	IRF4		
	SOCS1		

Table S5: Genes used to define cell state scores – lymphoid cells (related to Figures 4D - F)

Residency	Effector/Cytotoxicity	Exhaustion
CD69	IFNG	HAVCR2
ITGAE	GZMA	PDCD1
ITGA1	GZMB	CTLA4
CXCR6	GZMH	LAG3
DUSP6	PRF1	TIGIT
RGS1	CTSW	ENTPD1
CRTAM	CST7	CD244
PRDM1	NKG7	BTLA
RUNX3	GNLY	LAYN
ZNF683	KLRK1	CD160
	KLRB1	BATF
	KLRD1	TOX
	HLA-DRB1	
	HLA-DPA1	
	EOMES	
	TNFSF9	

Table S6: List of antibodies and reagents used in this study

Antibodies	Description	Company	Catalog No.	Working oncentration	Experiment used	RRID number
CD45	Rabbit polyclonal anti-CD45	abcam	ab10558	1:100	IF	AB_442810
CCL4	Rabbit polyclonal anti-CCL4	Invitrogen	PA5-114961	1:200	IF	AB_2899597
CXCL13	Goat polyclonal anti-CXCL13	R&D	AF801	1:200	IF	AB_355613
CD8	Mouse IgG2b anti-CD8	BioRad	MCA1817T	1:100	IF	AB_323534
CD3	CoraLite® Plus 488-conjugated Mouse IgG2b anti-CD3	ProteinTech	CL488-60181	1:100	IF	AB_2883113
IgG1 kappa Isotype Control (P3.6.2.8.1)	Alexa Fluor™ 488-conjugated Mouse IgG1,kappa	eBioscience	53-4714-80	1:100	IF	AB_470230
Anti-Mouse IgG (H+L)	Cy™3 AffiniPure® Donkey Anti-Mouse polyclonal	Jackson Immunoresearch	715-165-150	1:100	IF	AB_2340813
Anti-Rabbit IgG (H+L)	Alexa Fluor® 594 AffiniPure® Donkey Anti-Rabbit polyclonal	Jackson Immunoresearch	711-585-1521	1:100	IF	AB_2340621
Anti-Goat IgG (H+L)	Alexa Fluor® 647 AffiniPure® Donkey Anti-Goat polyclonal	Jackson Immunoresearch	705-605-003	1:100	IF	AB_2340436
DAPI		ThermoScientific	62248	1:2000	IF	NA
CD8	Anti-Hu CD8(C8/144B)-BX026 - Atto 550	Akoya Biosciences	4250012	1:100	CODEX	AB_2915960
TOX	Anti-Hu TOX(AKYP0098)-BX060 - Atto 550	Akoya Biosciences	240126	1:100	CODEX	NA
LAG3	Anti-Hu LAG3(AKYP0089)-BX055 - Alexa Fluor™ 647	Akoya Biosciences	4550058	1:100	CODEX	AB_3096409
IFNG	Anti-Hu IFNG(AKYP0093)-BX020 - Atto 550	Akoya Biosciences	4250062	1:100	CODEX	AB_3476455
CD4	Anti-Hu CD4(AKYP0048)-BX003 - Alexa Fluor™ 647	Akoya Biosciences	4550112	1:100	CODEX	AB_3094499
FOXP3	ANTI-HU FOXP3(AKYP0102)-BX031 - Alexa Fluor™ 647	Akoya Biosciences	4550071	1:100	CODEX	AB_2927679
TIGIT	Mouse IgG2a-BX002 - Atto 550	BioLegend	A15153G	1:100	CODEX	NA
TIM3	Goat polyclonal-BX030 - Cy5	R&D	AF2365	1:50	CODEX	AB_355235
CD45	Brilliant Violet 785™ anti-human CD45	Biolegend	304048	1:100	Flow cytometry	AB_2563129
CD3	Alexa Fluor® 700 Mouse Anti-Human CD3	BD Bioscience	557943	1:200	Flow cytometry	AB_396952
CD4	PerCP-Cyanine5.5 mouse anti-human CD4 (OKT4)	eBioscience	45-0048-42	1:100	Flow cytometry	AB_10804390
CD8	BV510 Mouse Anti-Human CD8	BD Bioscience	563256	1:200	Flow cytometry	AB_2738101

Reagents		Company	Catalog No.
Collagenase P		Millipore Sigma	11213857001
Deoxyribonuclease I from bovine pancreas		Sigma- Aldrich	DN25-1G
gentleMACS™ C Tubes		Miltenyi Biotech	130-093-237
Falcon™ Cell Strainers 40 µm		Fischer scientific	08-771-1
RPMI-1640		Thermo Scientific	61870127
Fetal Bovine Serum		ThermoFischer Scientific	A5256801
Trypan Blue		Invitrogen	T10282
ACK Lysing buffer		KD Medical	RGC-3015
Xylene substitute		Sigma- Aldrich	A5597-1GAL
Tris Base		Sigma- Aldrich	T1378-500G
EDTA		KD Medical	RGF-3130
Tween 20		Affymetrix	9005-64-5
Fluoro-Gel (with Tris Buffer)		Electron Microscopy Sciences	17985-10
UltraComp eBeads		ThermoFischer Scientific	01-2222-42
Zombie UV Fixable Viability Kit		Invitrogen	423108
Human TruStain FcX™		Biolegend	422302
Brilliant Stain Buffer		BD Biosciences	566349
Paraformaldehyde 16% Aqueous Solution EM Grade		Electron Microscopy Sciences	15710
Poly L-Lysine		Sigma- Aldrich	P8920-100ML
CODEX Conjugation Kit		Akoya Biosciences	7000009
CODEX Staining Kit		Akoya Biosciences	7000008
10X CODEX Buffer		Akoya Biosciences	7000001
CODEX Assay Reagent		Akoya Biosciences	7000002
Storage Buffer, 120mL		Akoya Biosciences	232107
96 well plates		Akoya Biosciences	7000006
CODEX Nuclear Stain		Akoya Biosciences	7000003
Codex Gaskets v2, pack of 10		Akoya Biosciences	7000010
96 well plate seals		Akoya Biosciences	7000007
coverslips		Akoya Biosciences	7000005
BX030 - Cy5-RX030		Akoya Biosciences	5350005
BX002 - Atto 550-RX002		Akoya Biosciences	5450023
Histochoice clearing agent		VWR	H103-4L
Ethanol, 100% solution (200 Proof) single bottle		VWR	95041-462
